# Error patterns on a computerized version of Raven’s progressive matrices in specific learning disorders

**DOI:** 10.3389/fpsyg.2026.1800128

**Published:** 2026-05-07

**Authors:** Matilde Spinoso, Mariagrazia Benassi, Alice Riccardi, Matteo Orsoni, Noemi Mazzoni, Andrea Brancaccio, Ottavia M. Epifania, Mariella Allegretti, Michela Muccinelli, Chiara Novelli, Debora de Chiusole, Pasquale Anselmi, Luca Stefanutti, Alice Bacherini, Irene Pierluigi, Giulia Balboni, Sara Giovagnoli

**Affiliations:** 1 Department of Psychology “Renzo Canestrari”, Cesena Campus, University of Bologna, Cesena, Italy; 2 Department of Theoretical and Applied Sciences, Faculty of Psychology, eCampus University, Novedrate, Italy; 3 Department of Philosophy, Sociology, Education, and Applied Psychology, University of Padua, Padova, Italy; 4 School of Psychology, University of East Anglia, Lawrence Stenhouse Building, Norwich, United Kingdom; 5 Department of Psychology and Cognitive Sciences, University of Trento, Rovereto, Italy; 6 Azienda Unità Sanitaria Locale (AUSL) della Romagna, Ravenna, Emilia-Romagna, Italy; 7 Department of Philosophy, Social Sciences and Education, University of Perugia, Perugia, Italy

**Keywords:** computerized assessment, error analysis, error patterns, fluid abilities, fluid intelligence, fluid reasoning, Raven’s matrices, specific learning disorder

## Abstract

Specific Learning Disorders (SLDs) are neurodevelopmental conditions characterized by persistent difficulties in reading, writing, or mathematics. These difficulties occur in the absence of intellectual disabilities, sensory impairments (visual/auditory), or neurological issues, indicating that SLDs do not reflect a lack of ability or a global cognitive impairment. Rather, they represent a different learning profile, where domain-specific difficulties coexist with otherwise adequate intellectual functioning. Consistent with this perspective and contemporary models conceptualizing intelligence as a multidimensional construct, research reveals marked heterogeneity in SLD cognitive profiles, particularly subtle weaknesses in working memory and processing speed, closely intertwined with Fluid Intelligence (FI). FI is defined as the ability to think logically, identify patterns, and solve novel problems independently of prior knowledge. Existing research has not found different FI levels in SLDs; however, most studies focused exclusively on quantitative measures, such as total accuracy, which may hinder individual differences in cognitive processes. Qualitative dimensions of FI, particularly error patterns, remain largely unexplored in this population, yet examining these patterns could reveal inefficiencies in reasoning that quantitative scores cannot capture. The present study investigated FI in children and adolescents with SLD using MatriKS, a new computerized version of Raven’s Progressive Matrices. A sample of 160 participants aged 7–19 years (88 with SLD, 72 typically developing) completed MatriKS alongside standardized cognitive and academic assessments. Among participants with SLD, further distinction was considered, based on academic impairment area (reading/writing disorder vs. mathematical disorder, isolated or combined). Results demonstrated good convergent validity of MatriKS with traditional Raven’s Matrices, and revealed significant group differences in overall accuracy, with the mathematics subgroup showing the most pronounced difficulties. Qualitative error analysis uncovered distinct patterns across SLD subtypes and developmental stages, with specific error types successfully distinguishing between diagnostic groups at different ages. These findings show that while global FI scores may fall within average range, qualitative error analysis can uncover specific inefficiencies, differentiating SLD subtypes across development. Therefore, incorporating error analysis into cognitive assessment could enhance the understanding of individual cognitive profiles and provide insights for interventions addressing underlying processing vulnerabilities, rather than solely academic skill deficits.

## 1 Introduction

Specific Learning Disorders (SLDs) are neurodevelopmental conditions characterized by persistent difficulties in reading, writing, or mathematics. These difficulties occur in the absence of intellectual disabilities, sensory impairments (vision/hearing), or neurological issues. Indeed, following the DSM-5 (Diagnostic and Statistical Manual of Mental Disorders, 5th edition; American Psychiatric Association, 2013) and the ICD-10 (International Classification of Diseases, 10th revision; World Health Organization, 1992) diagnostic criteria, individuals with SLDs have average global intellectual functioning. Nonetheless, intelligence is multidimensional and comprises several interrelated components. Thus, numerous studies (e.g., Cornoldi et al., 2014, 2019a, 2019b; De Clercq-Quaegebeur et al., 2010; Giofrè and Cornoldi, 2015; Iaia et al., 2025; Poletti, 2016; Scorza et al., 2023; Toffalini et al., 2017) have investigated whether specific cognitive profiles or patterns can be identified in individuals with SLD (see below). Among the various dimensions of intelligence that are crucially involved in learning, a core component is Fluid Intelligence (FI), which refers to the ability to reason logically and solve problems in novel situations, independently of prior knowledge (Cattell and Cattell, 1987). According to Cattell’s Investment Theory (Cattell, 1963, 1971, 1987), FI contributes to the development of crystallized intelligence and to the acquisition of academic skills. Several studies have supported this claim, showing that FI predicts achievement in various academic domains, including reading and mathematics (e.g., Green et al., 2017; Johann et al., 2020; Passolunghi et al., 2022; Peng et al., 2019).

Despite the importance of FI for learning, few studies have investigated FI in neurodevelopmental disorders, with some showing similar fluid abilities compared to typically developing children, especially in autism (e.g., Danis et al., 2023), and others highlighting a poorer performance in fluid reasoning, as well as in other cognitive processes essential to learning, such as executive functions (Semrud-Clikeman, 2012; Tamm and Juranek, 2012; Thorell, 2007). Moreover, some studies regarding Attention-Deficit/Hyperactivity Disorder (ADHD; e.g., Droder et al., 2024; Mano et al., 2018) have found both direct and indirect effects of FI on phonemic decoding and word recognition, suggesting that reduced fluid reasoning abilities may hinder reading acquisition in this population.

However, evidence on the development of FI in children with learning disorders is lacking. A first insight into this topic comes from studies on the performance of individuals with SLD on intelligence tests, especially on the Wechsler Scales (e.g., WISC-IV [Wechsler, 2003]; WAIS-IV [Wechsler, 2008]). Indeed, the WISC-IV and WAIS-IV batteries are some of the most widely used tools for assessing intellectual functioning (Evers et al., 2012). They evaluate different dimensions of intelligence through the following indices: Verbal Comprehension Index (VCI), Perceptual Reasoning Index (PRI), Working Memory Index (WMI) and Processing Speed Index (PSI). Among them, the PRI mainly assesses non-verbal fluid abilities, as measured through its subtests such as Block Design or Matrix Reasoning. Most of the studies on SLDs (e.g., Cornoldi et al., 2014, 2019a; Iaia et al., 2025; Poletti, 2016; Scorza et al., 2023; Toffalini et al., 2017) have focused on the discrepancy between the General Ability Index (GAI; derived from VCI + PRI) and the Cognitive Proficiency Index (CPI; derived from WMI + PSI), showing that, in both children and adults with SLD, the GAI index typically falls within the average range, whereas the CPI is usually lower than in controls. This discrepancy has been suggested as a hallmark of learning difficulties, which are characterized by average intellectual abilities, especially in the visuo-perceptual domain, and deficits in working memory and processing speed (Cornoldi et al., 2014, 2019a; Iaia et al., 2025; Poletti, 2016; Scorza et al., 2023; Toffalini et al., 2017).

However, SLDs are heterogeneous disorders, and individuals with learning difficulties frequently show considerable variability in their cognitive profiles (Cornoldi et al., 2019b; McGrath et al., 2019; Menghini et al., 2010; Pennington, 2006; Pennington et al., 2012; Poletti, 2016; Scorza et al., 2023; Toffalini et al., 2017; Willcutt et al., 2013). In line with this, Cornoldi et al. (2019b) identified two distinct subgroups among children with SLD based on WISC-IV profiles: a larger group showing lower Verbal Comprehension Index than Perceptual Reasoning Index, and another group showing the opposite pattern. Interestingly, children with SLD showing lower visuo-perceptual skills presented also weaker processing speed (Cornoldi et al., 2019b). This is not surprising, given that processing speed, as well as working memory, has been shown to be closely related to FI (Fry and Hale, 1996, 2000; Rodriguez-Martínez et al., 2023). Interesting results have also emerged when considering the specific domain of academic impairment (e.g., Poletti, 2016; Scorza et al., 2023; Toffalini et al., 2017). For instance, Toffalini et al. (2017), by comparing the WISC-IV profiles of children with different subtypes of SLDs (i.e., reading, spelling, arithmetical, and mixed disorder, based on the ICD-10 classification; World Health Organization, 1992, cited in Toffalini et al., 2017), found not only shared characteristics across the different disorders, such as the GAI-CPI discrepancy, but also partially distinct patterns upon further comparison of the four main indexes. In particular, by comparing the PRI and the VCI, they found that the PRI was higher than the VCI in children with reading disorder, lower than the VCI in those with arithmetical disorder, and similar to the VCI in children with spelling disorder; regarding mixed disorder, even though the PRI-VCI pattern did not significantly differ from that in reading disorder, it nonetheless exhibited the weakest overall intellectual profile compared to the other three groups (Toffalini et al., 2017). Similar differences in the WISC profiles, particularly between reading and mathematical disorders, have emerged in the study by Poletti (2016), who found that children with different subtypes of SLDs (based on the DSM-5 [American Psychiatric Association, 2013] and the Italian guidelines for SLD diagnosis [Consensus Conference, 2007; DSA, 2011, cited in Poletti, 2016]) showed different cognitive fragilities: those with mathematical impairment (isolated or combined with other learning impairments) had deficits not only in working memory and processing speed, but also in perceptual reasoning, whereas children with isolated reading impairment had deficits only in processing speed.

In addition to the Wechsler Scales, other widely used measures of FI are Raven’s Matrices, including Raven’s Progressive Matrices (RPM; Raven and Raven, 1938) and the Coloured Progressive Matrices (CPM; Raven, 1947, 1962), which require completing visual patterns by selecting the correct element from a series of response options denoted as distractors. Given their non-verbal format and their shorter administration time compared to the WISC-IV, Raven’s Matrices have been suggested as a valid alternative for the evaluation of intellectual abilities in clinical conditions, such as Intellectual Disability (Mungkhetklang et al., 2016), Autism (Nader et al., 2016) and Cerebral Palsy (Pueyo et al., 2008). This has also been suggested in relation to SLDs, given that several WISC-IV indices may sometimes be biased in children with learning difficulties (Giofrè and Cornoldi, 2015).

However, studies investigating fluid abilities in children and adolescents with SLD using Raven’s Matrices are lacking. In a study by Bayliss et al. (2005) investigating working memory abilities in children with learning difficulties, participants also completed the CPM, and it emerged that children with learning difficulties showed a non-significant trend for a higher accuracy on Raven’s Matrices compared to typically developing peers. However, more studies are needed to reach consistent conclusions on whether performance on this type of task may differ in children with SLD compared to typically developing peers. Moreover, when evaluating FI with Raven’s Matrices, it has been argued that, in addition to focusing on the overall accuracy score (i.e., number of correct answers), a qualitative analysis, such as the analysis of error patterns, could add important information by shedding light on the problem-solving strategies and, thus, on the underlying cognitive processes involved in the resolution, which may vary depending on age or in presence of cognitive impairments (see Kunda et al., 2016, for an extensive discussion).

The importance of a qualitative analysis of the performance on Raven’s Matrices has been highlighted by Raven himself, who, in his test manuals (Raven et al., 2003), classified possible errors into four categories: Repetition (R, i.e., the chosen distractor mirrors a matrix piece located next to the blank space), Difference (D, i.e., the chosen distractor is qualitatively distinct from the other alternatives, for example by being blank or containing elements not found elsewhere in the matrix), Wrong Principle (WP, i.e., the chosen distractor replicates or combines elements from different parts of the matrix according to an incorrect rule, due to a misinterpretation of the underlying relationships), and Incomplete Correlate (IC, i.e., the chosen distractor closely approximates the correct answer but is not entirely accurate; see Kunda et al., 2016; Epifania et al., 2026). Given the characteristics of the matrix, such as its dimensionality, it might happen that distractors that are normally identified as R present the same characteristics as the IC distractors. As such, a new category should be considered, denoted as Repetition/Incomplete Correlate (R/IC; see Epifania et al., 2026).

In line with that, several studies have investigated the possible presence of specific error patterns on Raven’s Matrices, in both clinical and non-clinical samples. For instance, Babcock (2002) found that adults with high versus low ability (based on correct answers) made different types of errors, with the most frequent error in high ability adults being IC, while no specific error pattern was found in low ability participants.

Research on clinical populations has yielded similar results, identifying specific error patterns in children with neurodevelopmental disorders, such as Down Syndrome (Belacchi et al., 2012; Gunn and Jarrold, 2004), Cerebral Palsy (Asano et al., 2023), and Intellectual Disability (Goharpey et al., 2013). These findings suggest that analyzing error types can provide valuable information about problem-solving strategies and logical reasoning in these populations. However, studies investigating error patterns on Raven’s Matrices among children with SLD are still lacking,

Research has also explored age-related changes in performance on Raven’s Matrices. Among the general population, most studies on adults have shown that older individuals tend to perform more poorly on Raven’s Matrices compared to younger ones (e.g., Panek and Stoner, 1980); conversely, performance among children and adolescents tends to improve with age (e.g., Gunn and Jarrold, 2004). This is in line with theories of FI development, which state that it develops rapidly during childhood, continues to increase during adolescence through early adulthood, before starting to decline gradually in adulthood and older age (Fry and Hale, 2000; McArdle et al., 2002).

Regarding error patterns, a few studies have investigated the potential effect of age. While Babcock (2002) found no age-related differences in error types among adults, Gunn and Jarrold (2004) showed that, in their sample of typically developing children, the proportion and distribution of error types changed in relation to age, with older children making more R and IC errors and less D and Individuation (i.e., WP; Kunda et al., 2016) errors than younger children. In contrast, among individuals with Down Syndrome, they found a positive correlation between age and performance, but no significant differences in the error pattern across age (Gunn and Jarrold, 2004).

Studies on age-related differences in Raven’s Matrices performance among children with SLD are currently lacking. However, there is evidence that FI development is closely linked to the development of working memory and processing speed (e.g., Fry and Hale, 1996, 2000), two cognitive functions that appear to be often impaired in children with learning disorders (e.g., Cornoldi et al., 2014, 2019a; De Clercq-Quaegebeur et al., 2010; Poletti, 2016; Toffalini et al., 2017). Therefore, examining potential age-related changes in the performance on Raven’s Matrices in SLDs, both quantitatively and qualitatively, seems warranted.

In conclusion, the reviewed evidence highlights the importance of assessing fluid abilities in children with SLD, as they may display different levels of FI development, which can play a role in learning as either a protective factor or a risk factor: when efficient, FI could serve as a strength that supports the development of scholastic skills (Cattell, 1971, 1987; Green et al., 2017; Johann et al., 2020; Passolunghi et al., 2022; Peng et al., 2019); whereas when inefficient or poor, it may represent a vulnerability that hinders learning progress (Droder et al., 2024; Mano et al., 2018). Moreover, when assessing FI with Raven’s Matrices, going beyond the overall accuracy score and adding a qualitative error analysis could offer deeper insight into the cognitive functioning of children with SLD (Asano et al., 2023; Belacchi et al., 2012; Goharpey et al., 2013; Gunn and Jarrold, 2004; Kunda et al., 2016). In this context, recently developed computerized versions of the Raven’s Matrices (e.g., MatriKS; de Chiusole et al., 2024; de Chiusole et al., 2025) may provide a more efficient and information-rich alternative solution compared to the paper-and-pencil format, as they automatically record a large amount of detailed information on the test-taker’s performance, making data collection easier and more time efficient (Mead and Drasgow, 1993; Witt et al., 2013).

Based on these considerations, the present study aimed to investigate the advantages of analyzing the types of errors in FI tasks in children with SLDs using the new digital assessment tool MatriKS, which administers Raven’s like matrices (de Chiusole et al., 2024; Anselmi et al., 2025). Specifically, the following objectives were addressed:

1) To examine the convergent validity of MatriKS in children with SLD by analyzing the correlations between performance on MatriKS (total accuracy score) and raw scores on Raven’s Progressive Matrices, both measuring fluid intelligence. In particular, we aimed to analyze the correlations between MatriKS 4–11 and Raven’s CPM, and between MatriKS 12 + and Raven’s SPM (Standard Progressive Matrices).
2) To compare the total accuracy score obtained in MatriKS 4–11 and 12 + between healthy controls and participants with SLD, considering the effect of age on these measures (age is included as a covariate) and observing the distribution of the total number of errors.
3) To investigate the presence of differences in the proportion of errors (i.e., R, WP, D, IC, R/IC) between participants with SLD and the control group, separately for MatriKS 4–11 and MatriKS 12 + .
4) To determine whether specific error patterns (R, WP, D, IC, R/IC) on MatriKS 4–11 and 12 + can serve as cognitive markers for distinguishing between diagnostic groups by conducting multinomial logistic regression analyses. Specifically, with this analysis we aimed to assess whether the frequency and distribution of error types on MatriKS predict the probability of belonging to different SLD subtypes (see more details in the participants section) versus the control group, thereby providing insights into the diagnostic utility of qualitative error analysis in clinical assessment.

## 2 Materials and methods

### 2.1 Instruments

#### 2.1.1 MatriKS

MatriKS (de Chiusole et al., 2024; de Chiusole et al., 2025) is a computerized test, built on the characteristics and principles of Raven’s Matrices, and developed to assess FI in individuals of different ages (i.e., children, adolescents, and adults), with or without clinical conditions. As traditional Raven-like tasks, it is composed of a series of visual patterns, each containing a missing element that must be completed by selecting one of several alternatives, with the incorrect options also termed “distractors.”

Two versions of MatriKS are available: MatriKS 4–11 for children aged four to 11 years old, and MatriKS 12 + for adolescents and adults aged 12 and over. In both versions, each stimulus consists of two parts. One is the matrix, which can be of three types: monothematic matrix (a single figure containing a missing piece), 2×2 matrix (a set of four cells with one missing), or 3×3 matrix (a set of nine cells with one missing). The matrix appears on the left of the screen and has the missing cell in its low-right corner. The other part of the stimuli consists of the response options, which are five or eight, depending on the type of matrix, and includes the correct response and the distractors. The response options, as well as the matrices, were created with the matRiks package, which formalizes and implements the definition of the error categories (see Introduction) for the generation of the distractors (Brancaccio et al., 2025). As such, it is possible to collect information on the error patterns that are consistent with the literature. In addition to these error types (i.e., R, D, WP, and IC), in the present study another error category, referred to as R/IC, was considered, which includes distractors that exhibit characteristics of both R and IC errors. In MatriKS, the response options are shown next to the corresponding matrix, on the right of the screen. The system records the participant’s selected answer (i.e., correct, incorrect, non-provided), the selected distractor in case of an incorrect response, the time spent on the whole test and on each problem, and the average time per item.

MatriKS 4–11 includes 38 stimuli, of which 21 are colored and 17 are black and white. As for the dimensions, four matrices are monothematic, 19 matrices are 2×2, and 15 matrices are 3×3. The monothematic and 2×2 matrices include five response options, while the 3×3 matrices include eight response options. To be solved, the stimuli of MatriKS 4–11 require skills related to visual perception, elaboration of general configuration, reasoning and elementary inference. MatriKS 12 + includes 45 stimuli, each with a dimension of 3×3 and eight response options, except for three stimuli with a dimension of 2×2 and five response options. The two versions have 11 stimuli in common. To be solved, the stimuli of MatriKS 12 + requires the skills involved in MatriKS 4–11, plus advanced skills of reasoning and logic inference.

MatriKS was administered to each child individually by psychologists or trained researchers. The test was presented on the PsycAssist platform (de Chiusole et al., 2024) available at https://psycassist.fisppa.unipd.it by using a tablet (IoS operating system with a 10.9in screen). The administration of MatriKS was carried out with the tablet held horizontally. The MatriKS test was introduced by a brief animated video (95 s), which presented the test, explained the structure of the stimuli and the correct way to select the response from the response list. After the video presentation, children were presented with four practice items, and then the test started. There were no time constraints. A response was registered once the child touched one of the response options. If the child struggled to find an answer to an item, the researcher could decide to skip it. The MatriKS system automatically records omissions as “skipped responses,” which may reflect perceived task difficulty or fatigue. No feedback was given about the correctness of the responses provided by the children. The stimuli were presented one at a time in random order.

#### 2.1.2 Fluid intelligence measures

The following standardized instruments were administered to assess fluid intelligence abilities according to the participant’s age and level of schooling. Both the instruments were administered individually in a quiet room, by trained psychologists ( n=2 ) or psychology interns ( n=9 ) in a counterbalanced order, for both types of tests (computerized vs. traditional, i.e., MatriKS vs. CPM/SPM). During the diagnostic assessment, for clinical reason, a subset of participants with SLD was administered the WISC-IV (Wechsler Intelligence Scale for Children-IV; Wechsler, 2003; Italian adaptation by Orsini et al., 2012) and WAIS-IV (Wechsler Adult Intelligence Scale-IV; Wechsler, 2008; Italian adaptation by Orsini and Pezzuti, 2013) instead of the paper-and-pencil version of Raven’s Progressive Matrices (CPM/SPM). Consequently, convergent validity analyses were conducted only on the subsample of participants with SLD for whom both MatriKS and the corresponding Raven’s measure were available with eight participants per age group excluded from these analyses.

The Colored Progressive Matrices (CPM; Raven, 1947, 1962; Italian adaptation of CPM by Belacchi et al., 2008): The CPM is a widely used measure of non-verbal reasoning for children and elders. It consists of 36 items of increasing difficulty, organized into three series (i.e., A, AB, and B) having 12 items, requiring the identification of the piece that best completes the matrix, choosing among 6 alternatives. Serie A evaluates the similarities detection (based on shape, dimension, direction, quantity, orientation, figure/background, density criteria), Serie AB assesses the symmetry detection, and Serie B measures the conceptual thinking (i.e., detection of abstract relations according to operant-deductive logic and their retention in working memory). Each correct answer scores one point, for a maximum total score of 36. In this study, the test was administered to children aged 7 to 11.11 years.

Raven’s Standard Progressive Matrices (SPM; Raven, 1938; Raven et al., 1989; Italian adaptation by Picone et al., 2018): The SPM measures non-verbal reasoning and are standardized for individuals aged 6 to 60 years. They consist of visual matrices with a missing element to be completed by selecting the correct option from distractors. The battery includes 60 items divided into five series (A–E), each composed of 12 items with increasing difficulty. Each series addresses distinct cognitive themes: continuous patterns (Series A), analogical relationships (Series B), progressive pattern transformations (Series C), figure permutations (Series D), and decomposition of figures into constituent parts (Series E; Burke, 1958). In this study, the test was administered to children aged 12 to 19.11 years.

Both MatriKS and the standard paper and pencil measure of fluid intelligence (CPM and SPM) were administered without time constraints. The instruments were administered in a counterbalanced order with respect to the type of test (computerized vs. traditional, i.e., MatriKS vs. CPM/SPM). Moreover, between the administration of MatriKS and the traditional instruments occurred 3 to 4 weeks.

### 2.2 Participants

The sample consisted of 160 children and adolescents (89 females, 56%; 71 males, 44%) aged 7.8 to 19.11 years (M = 12.23, SD = 2.49), divided into two age groups based on the MatriKS test version administered. The younger age group (7.8–11.9 years) comprised 74 participants (M = 10.02, SD = 1.17; 42 females, 57%; 32 males, 43%). Of these, 36 were typically developing (TD) children (49%) and 38 (51%) had a diagnosis of SLD according to DSM-5 (American Psychiatric Association, 2013) and ICD-10 (World Health Organization, 1992) criteria. The older age group (12–19.11 years) comprised 86 participants (M = 14.08, SD = 1.67; 47 females, 55%; 39 males, 45%). Of these, 36 were TD adolescents (42%), and 50 (58%) met diagnostic criteria for SLD based on DSM-5 and ICD-10 classifications.

Overall, the sample included 72 TD participants (45%) and 88 participants with SLD (55%). Within the SLD group, participants were further divided into two subgroups, separately for MatriKS versions: (SLD-Dys) a reading/writing subgroup, including participants with dyslexia and/or spelling disorder, either isolated or combined; and (SLD-Mixed) a mathematics subgroup, including participants with isolated dyscalculia or mixed disorders involving dyscalculia and dyslexia and/or spelling disorder.

Table 1 reports the descriptive statistics of the total sample of participants (N = 160), divided into two groups based on age and the version of the MatriKS test administered: MatriKS 4–11 years (*N* = 74) and MatriKS 12 + years (*N* = 86). Within each group, participants with TD and those with SLD are distinguished. In addition, the table includes the descriptive data for the two SLD subgroups, that is, SLD-Dys (reading and/or writing disorders) and SLD-Mixed (isolated dyscalculia or mixed disorders involving dyscalculia and dyslexia and/or spelling disorder), to provide a more detailed overview of the sample characteristics.

**Table 1:** Descriptive statistics of the total sample (*N* = 160), divided into two groups based on the MatriKS version administered (MatriKS 4–11 and MatriKS 12+).

Groups	MatriKS 4–11		MatriKS 12+	
	n (% total)	Age in years, M (SD)	n (% total)	Age in years, M (SD)
TD	36 (49%)	10.18 (1.24)	36 (42%)	13.73 (1.29)
SLD	38 (51%)	9.86 (1.09)	50 (58%)	14.33 (1.88)
SLD-Dys	21 (28%)	9.26 (0.96)	24 (28%)	14.14 (1.72)
SLD-Mixed	17 (23%)	10.61 (0.73)	26 (30%)	14.50 (2.03)
Total	74	10.02 (1.17)	86	14.08 (1.67)

Clinical articipants (SLD) were recruited from two primary sources: (1) the Child and Adolescent Neuropsychiatry Units (UONPIA) of Forlì-Cesena and Rimini (AUSL Romagna, Italy), and (2) the Clinical Service SPEV (Servizio di Potenziamento cognitivo dell’Età Evolutiva) of the Department of Psychology, University of Bologna (Italy). Clinical participants accessed these services for an initial assessment of suspected SLD, reassessment of previously diagnosed SLD, or were receiving ongoing treatment at these facilities. Control participants were recruited from schools located in different regions of Northern, Central, and Southern Italy, covering all educational levels from kindergarten to high school. Most control participants included in this study completed only MatriKS. Data collection for SLD was conducted at AUSL Romagna sites by licensed psychologists in collaboration with the principal investigators of the respective UONPIA sites and a research team from the Department of Psychology, University of Bologna. Data analysis was performed by the Laboratory of Psychometrics and Neuropsychology of the Department of Psychology, University of Bologna.

The inclusion criteria were as follows:

1. Age between 7.00 and 19.11 years.
2. For the SLD group: formal diagnosis of SLD according to DSM-5 (American Psychiatric Association, 2013) and ICD-10 (World Health Organization, 1992) criteria, established through comprehensive neuropsychological assessment.
3. For the TD group: absence of learning disorders, neurological conditions, or psychiatric diagnoses based on parent report and clinical history.
4. Adequate vision and hearing (normal or corrected-to-normal) to complete tablet-based tasks.
5. Ability to understand task instructions in Italian.

The exclusion criteria were as follows:

1. Motor, visual, or auditory impairments that would compromise the ability to perform tablet-based tasks or communicate responses to the examiner.
2. Intellectual disability, defined as Total Intelligence Quotient (TIQ) < 70 (more than 2 standard deviations below the mean, corresponding to the range of 55–69), as assessed by standardized cognitive batteries (WISC-IV, Orsini et al., 2012; WAIS-IV, Orsini and Pezzuti, 2013).
3. Primary diagnosis of other neurodevelopmental disorders (e.g., Autism Spectrum Disorder).
4. Primary diagnosis of emotional or behavioral disorders.
5. Comorbid psychiatric conditions.

The study was conducted in accordance with the Declaration of Helsinki (Williams, 2008) and approved by the Ethics Committee of Romagna (Comitato Etico di Area Vasta Romagna e IRST [CET–C. E. ROM]; protocol number 410/2025 I.5/46; approval date: July 9, 2025). Written informed consent was obtained from parents or legal guardians of all participants prior to enrollment in the study. Only participants for whom signed parental informed consent was available were included. Age-appropriate verbal consents were also obtained from all children and adolescent participants.

### 2.3 Data analysis

All statistical analyses were performed using IBM SPSS Statistics version 27 (IBM Corp, 2017) and JASP version 0.95.4 (JASP Team, 2025).

To address the study objectives, the following analyses were conducted. To examine the convergent validity of MatriKS (Objective 1), after verifying that skewness and kurtosis values indicated normal univariate distributions (Tabachnick and Fidell, 2013), Pearson’s parametric correlation analyses were conducted to assess the associations between total accuracy scores on MatriKS 4–11 and raw scores on the CPM, and between total accuracy scores on MatriKS 12 + and the SPM.

To compare overall accuracy between groups (Objective 2), two separate Analyses of Covariance (ANCOVAs) were conducted for the MatriKS 4–11 (7.8–11.9 years) and MatriKS 12 + (12–19.11 years) versions. In these models, total accuracy (number of correct items) was entered as the dependent variable, group (TD vs. SLD-Dys [reading/writing subgroup] vs. SLD-Mixed [mathematics subgroup]) as the independent variable, and age as a covariate. The distribution of the total number of errors across groups was also examined.

To investigate differences in error types between groups (Objective 3), separate Generalized Linear Models (GzLMs) were conducted for each error type proportion (R, WP, D, IC, and the combined R/IC category). These models allows analyzing differences between groups with respect to error rates that may even have values equal to or close to zero, making them very difficult to interpret with traditional methods based on the normal distribution. Analyses were performed separately for MatriKS 4–11 and MatriKS 12 + versions. Each model included group (TD vs. SLD-Dys vs. SLD-Mixed) as the independent variable and age as a covariate.

To determine whether error patterns could distinguish between diagnostic groups (Objective 4), multinomial logistic regression models were fitted to assess whether error type proportions (R, WP, D, IC, R/IC) could predict group membership (TD vs. SLD-Dys vs. SLD-Mixed). A Generalized Linear Model (GzLMs) with a normal distribution and identity link function was fitted. The dependent variable was predicted by the between-subjects factor groups (ascending order) and the continuous covariate age, including an intercept. Parameters were estimated via maximum likelihood, and significance was evaluated using Wald statistics with 95% confidence intervals. Estimated marginal means were computed for groups and pairwise comparisons were conducted using simple contrasts with LSD adjustment. The analysis examined the contribution of each error type in differentiating between the three groups.

## 3 Results

### 3.1 Convergent validity of MatriKS (objective 1)

Skewness and kurtosis analysis indicated normal univariate distributions, as all values were below |2| (Tabachnick and Fidell, 2013; see Tables 2, 3). Based on this, Pearson’s parametric correlation analyses) were conducted separately for the MatriKS 4–11 and MatriKS 12 + versions to examine their convergent validity by assessing their association with Raven’s Progressive Matrices raw scores (CPM and SPM, respectively). For the MatriKS 4–11 version, analyses were performed on a subgroup of children with SLD who completed both measures (*n* = 30), revealing a significant positive correlation between total accuracy and CPM raw scores, *r* = 0.50, *p* = 0.005, indicating moderate association that may be suggestive of convergent validity but should be interpreted with caution, as correlations in this range reflect only limited shared variance between measures (Post, 2016). For the MatriKS 12 + version, a separate analysis conducted on adolescents with SLD who completed both tests (*n* = 42) showed a significant positive correlation between total accuracy and SPM raw scores, *r* = 0.68, *p* < 0.001, indicating a stronger association that provide support of convergent validity in the older age group, although correlations in this range may still warrant cautious interpretation. Overall, these findings provide preliminary evidence suggestive of convergent validity across developmental stages. Evidence for convergent validity in a larger TD sample is reported in the full multimethod validation study of MatriKS 4–11 (de Chiusole et al., 2025).

**Table 2:** Descriptive statistics for MatriKS 4–11 and Raven CPM accuracy scores for the three groups (i.e., TD, SLD-Dys, SDL-Mixed).

Statistic	MatriKS 4–11			CPM	
	TD	SLD-Dys	SLD-Mixed	SLD-Dys	SLD-Mixed
Mean	32.89	27.24	28.18	28.41	29.23
Standard Deviation	3.85	6.15	6.18	3.74	3.59
Skewness	−0.80	−0.35	−0.83	0.39	0.71
Kurtosis	−0.06	0.15	0.67	−0.92	−0.84
N	36	21	17	17	13

CPM, Coloured Progressive Matrices; TD, Typically Developing; SLD-Dys, Specific Learning Disorder-reading/writing subgroup; SLD-Mixed, Specific Learning Disorder-mathematics subgroup.

**Table 3:** Descriptive statistics for MatriKS 12 + and Raven SPM accuracy scores for the three groups (i.e., TD, SLD-Dys, SDL-Mixed).

Statistic	MatriKS 12+			SPM	
	TD	SLD-Dys	SLD-Mixed	SLD-Dys	SLD-Mixed
Mean	34.44	31.83	25.85	43.27	36.35
Standard Deviation	8.24	7.22	9.04	6.9	7.07
Skewness	−1.08	−0.77	−0.65	−0.33	−0.48
Kurtosis	1.45	0.71	−0.46	0.02	0.05
N	36	24	26	22	20

SPM, Standard Progressive Matrices; TD, Typically Developing; SLD-Dys, Specific Learning Disorder-reading/writing subgroup; SLD-Mixed, Specific Learning Disorder-mathematics subgroup.

### 3.2 Differences in MatriKS total accuracy among groups (objective 2)

To examine group differences in overall performance on the MatriKS tasks, the Analyses of Covariance were conducted for the 4–11 and 12 + versions separately, including age as a covariate.

**Figure 1:**
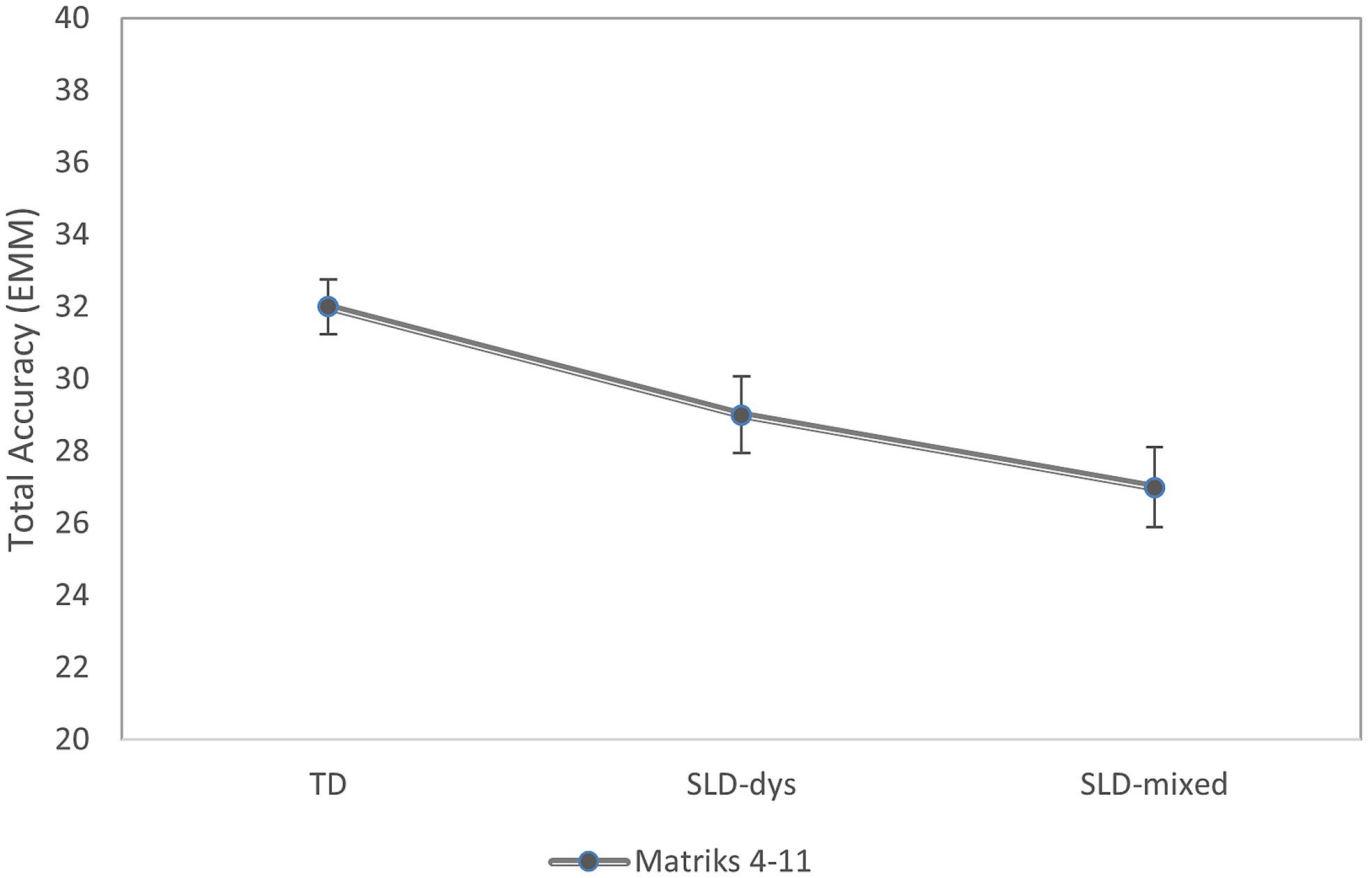
Estimated marginal means (EMMs) of total accuracy by diagnostic group in MatriKS 4–11. The *y*-axis represents the estimated mean of total accuracy, and the *x*-axis shows the SLD-Dys and SLD-mixed groups. Points indicate EMMs, and vertical bars represent standard error.

For the MatriKS 4–11 version, the analysis revealed a significant main effect of group on total accuracy, *F*(2, 70) = 10.22, *p* < 0.001, η^2^_p_ = 0.23, indicating a large effect size (Cohen, 2013). After adjusting for age, control children showed higher accuracy (M = 32.51; SE = 0.76) than both children with SLD-Dys (M = 29.01; SE = 1.06) and those in the SLD-Mixed group (M = 26.79; SE = 1.14); see Figure 1. Bonferroni-corrected comparisons indicated that the control group performed significantly better than the SLD-Dys group (mean difference = 3.50, *p* = 0.031) and the SLD-Mixed group (mean difference = 5.72, *p* < 0.001), whereas the SLD-Dys and SLD-Mixed groups did not differ significantly (*p* = 0.530). Age effect was significant *F*(1, 70) = 21.78, *p* < 0.001, indicating that higher age was associated with higher accuracy (b = 2.35; 95% CI [1.34,3.35]).

**Figure 2:**
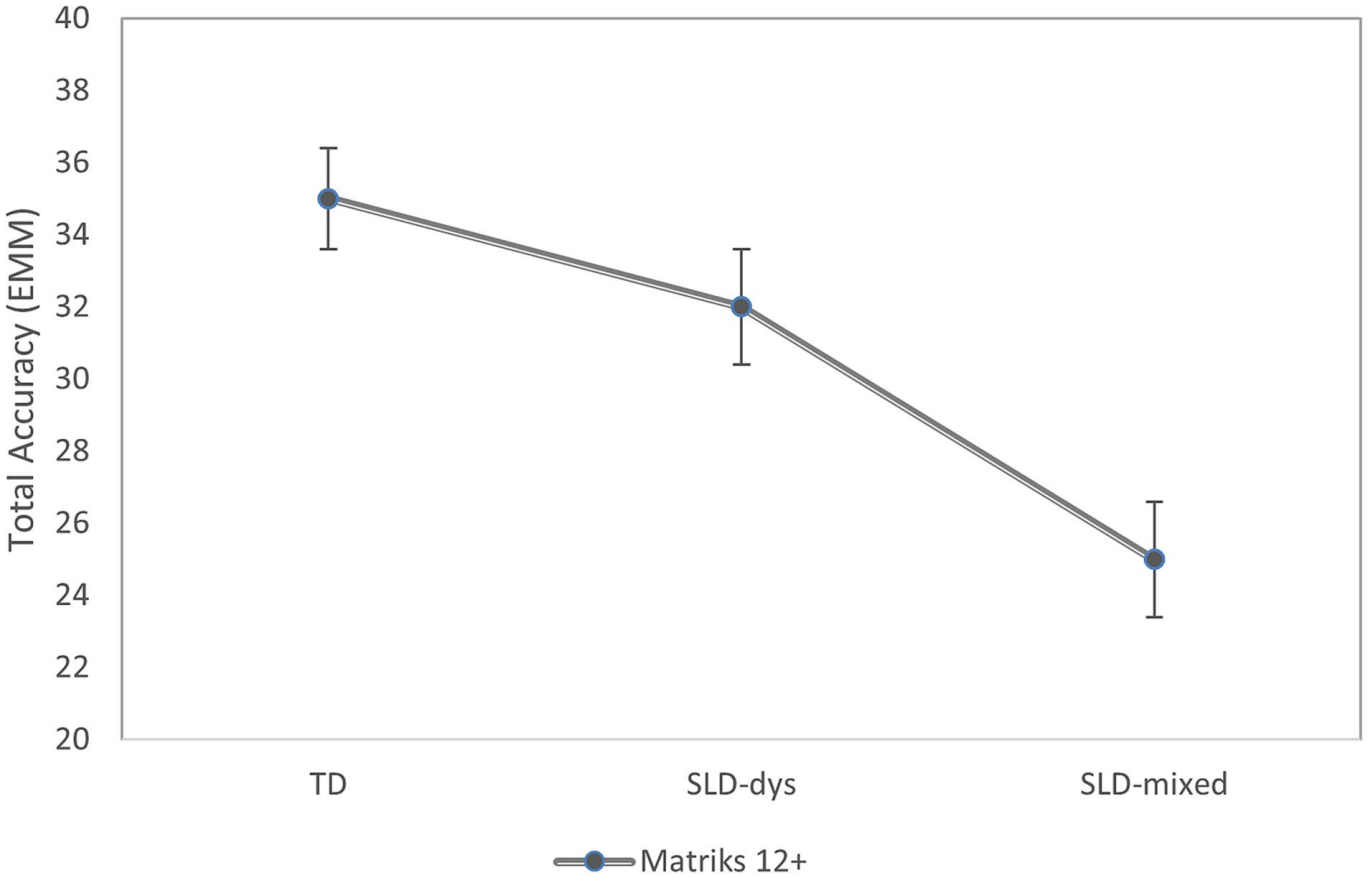
Estimated marginal means (EMMs) of total accuracy by diagnostic group in MatriKS 12+. The y-axis represents the estimated mean of total accuracy, and the x-axis shows the diagnostic groups. Points indicate EMMs, and vertical bars represent standard error.

For the MatriKS 12 + version, the ANCOVA also showed a significant main effect of group, *F*(2, 82) = 10.43, *p* < 0.001, η^2^_p_ = 0.20, indicating a large effect size (Cohen, 2013). Adjusted means indicated the highest accuracy in the control group (M = 34.87; SE = 1.35), followed by adolescents with SLD-Dys (M = 31.75; SE = 1.64), and the SLD-Mixed group (M = 25.32; SE = 1.59). Pairwise comparisons showed that controls scored significantly higher than the SLD-Mixed group (mean difference = 9.56, *p* < 0.001), but did not differ significantly from the SLD-Dys group (*p* = 0.233); see Figure 2. Additionally, adolescents with SLD-Dys performed significantly better than those in the SLD-Mixed group (mean difference = 6.43, *p* = 0.018). In contrast to the younger cohort, age was not a significant covariate in the 12 + model, *F*(1, 82) = 3.33, *p* = 0.072 (b = 1.25; 95% CI [0.18, 2.29]).

Together, these results indicate group differences in total accuracy for both versions of the task, with a consistent pattern in which control participants outperform those with SLD-Dys and SLD-Mixed profiles, although the specific contrasts vary across developmental stages.

### 3.3 Differences in error patterns among groups (objective 3)

To investigate potential differences in error patterns among groups (i.e., TD, SLD-Dys and SLD-Mixed), different approaches have been applied, and the obtained solutions have been compared.

Five separate GzLMs were conducted for each error category (i.e., R, WP, D, IC, and the combined R/IC category).

In each version of MatriKS and for each participant, error type proportions were calculated by dividing the frequency of each specific error type committed in all the tasks by the total number of possibilities to commit that error type across all MatriKS items. This approach was necessary because some matrices contain multiple distractors belonging to the same error category. Analyses were performed separately for MatriKS 4–11 and MatriKS 12 + versions. Each model included Group (TD vs. SLD-Dys vs. SLD-Mixed) as the independent variable and Age as a covariate.

#### 3.3.1 Error pattern in MatriKS 4–11 version

**Figure 3:**
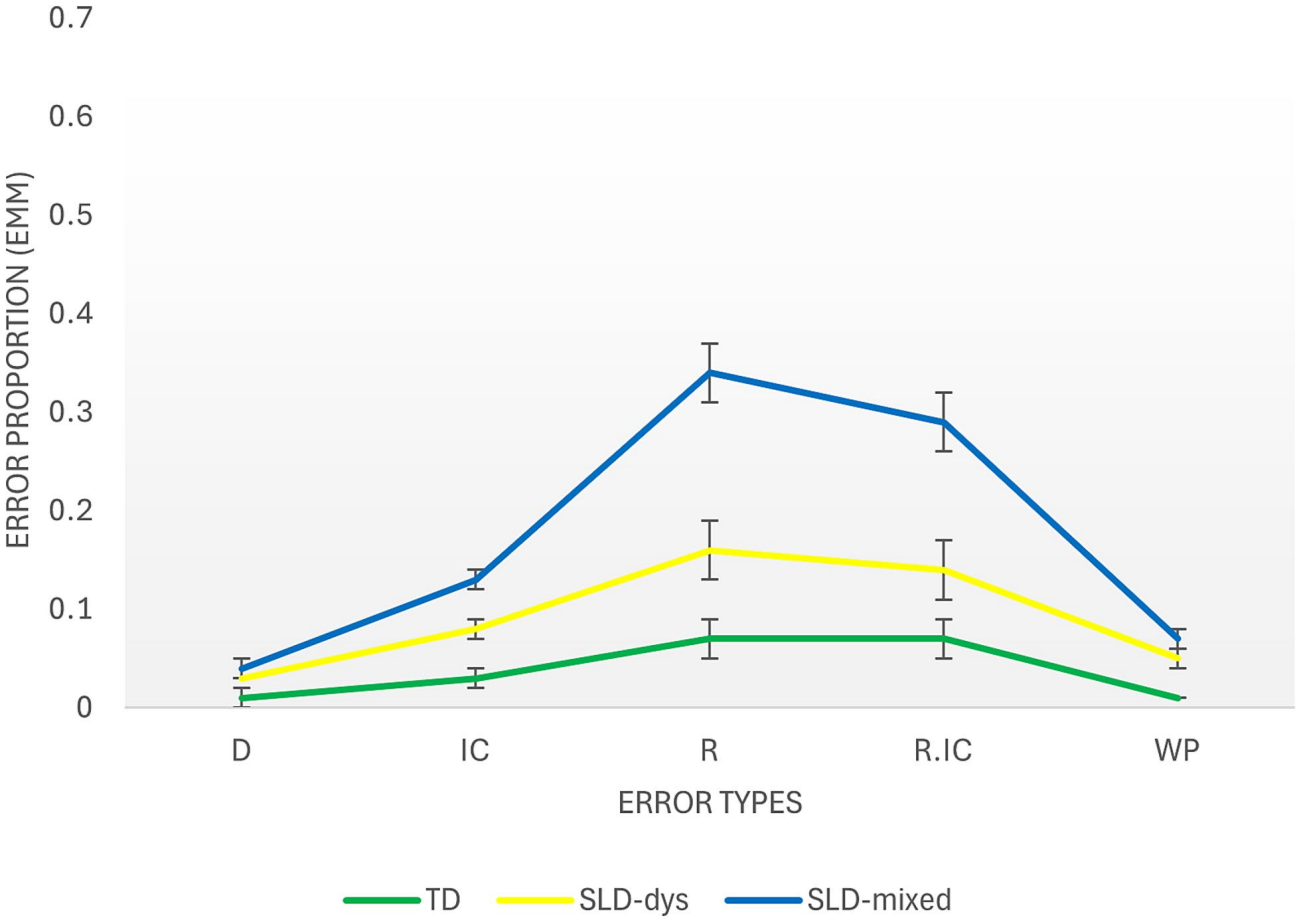
Analysis of error types in TD and SLD participants in the MatriKS 4–11 version. The *x*-axis represents the different error types, and the *y*-axis shows the average percentage of error proportion in each group. TD, typically developing; SLD-Dys, specific learning disorder-reading/writing subgroup; SLD-Mixed, specific learning disorder-mathematics subgroup; M, mean; SE, standard error.

The differences between groups in the proportion of error types in the MatriKS 4–11 version are illustrated in Figure 3. Concerning the R error, the analysis revealed a significant main effect of Group (Wald χ^2^(2) = 11.43, *p* = 0.003) and a significant effect of Age (Wald χ^2^(1) = 17.44, *p* < 0.001), indicating that older children made fewer repetition errors (b = −0.05; 95% CI [−0.08, −0.03]). Pairwise comparisons showed that the SLD-Mixed group (M = 0.180; SE = 0.03) produced significantly more R errors than both the TD group (M = 0.068; SE = 0.02 Wald χ^2^(1) = 11.42, *p* < 0.001) and the SLD-Dys group (M = 0.093; SE = 0.02, Wald χ^2^(1) = 4.74, *p* = 0.029). The TD and SLD-Dys groups did not differ significantly (*p* = 0.440).

A significant main effect of Group also emerged for WP errors (Wald χ^2^(2) = 12.50, *p* = 0.002), indicating that the SLD-Dys group made more WP errors (M = 0.039; SE = 0.01) than the TD group (M = 0.010; SE = 0.00; Wald χ^2^(1) = 11.86, *p* < 0.001). The SLD-Mixed group (M = 0.025; SE = 0.01) did not differ significantly from either the TD group (*p* = 0.093) or the SLD-Dys group (*p* = 0.157).

No significant main effect of Group was found for D error proportions (Wald χ^2^(2) = 2.72, *p* = 0.257) and for the IC error (Wald χ^2^(2) = 4.68, *p* = 0.096), with groups having similar patterns. Age was not a significant covariate (*p* = 0.332).

A significant main effect of Group was also found for the R/IC category (Wald χ^2^(2) = 6.19, *p* = 0.045). In details, the SLD-Mixed group (M = 0.151; SE = 0.03) produced significantly more R/IC errors than the TD group (M = 0.069; SE = 0.02, Wald χ^2^(1) = 5.76, *p* = 0.016) and the SLD-Dys group (M = 0.069; SE = 0.03, Wald χ^2^(1) = 3.98, *p* = 0.046). The TD and SLD-Dys groups did not differ (*p* = 0.999). Age had a significant effect (Wald χ^2^(1) = 19.45, *p* < 0.001), indicating that older children made fewer R/IC errors (b = −0.06; 95% CI [−0.08, −0.03]).

#### 3.3.2 Error patterns in MatriKS 12 + version

**Figure 4:**
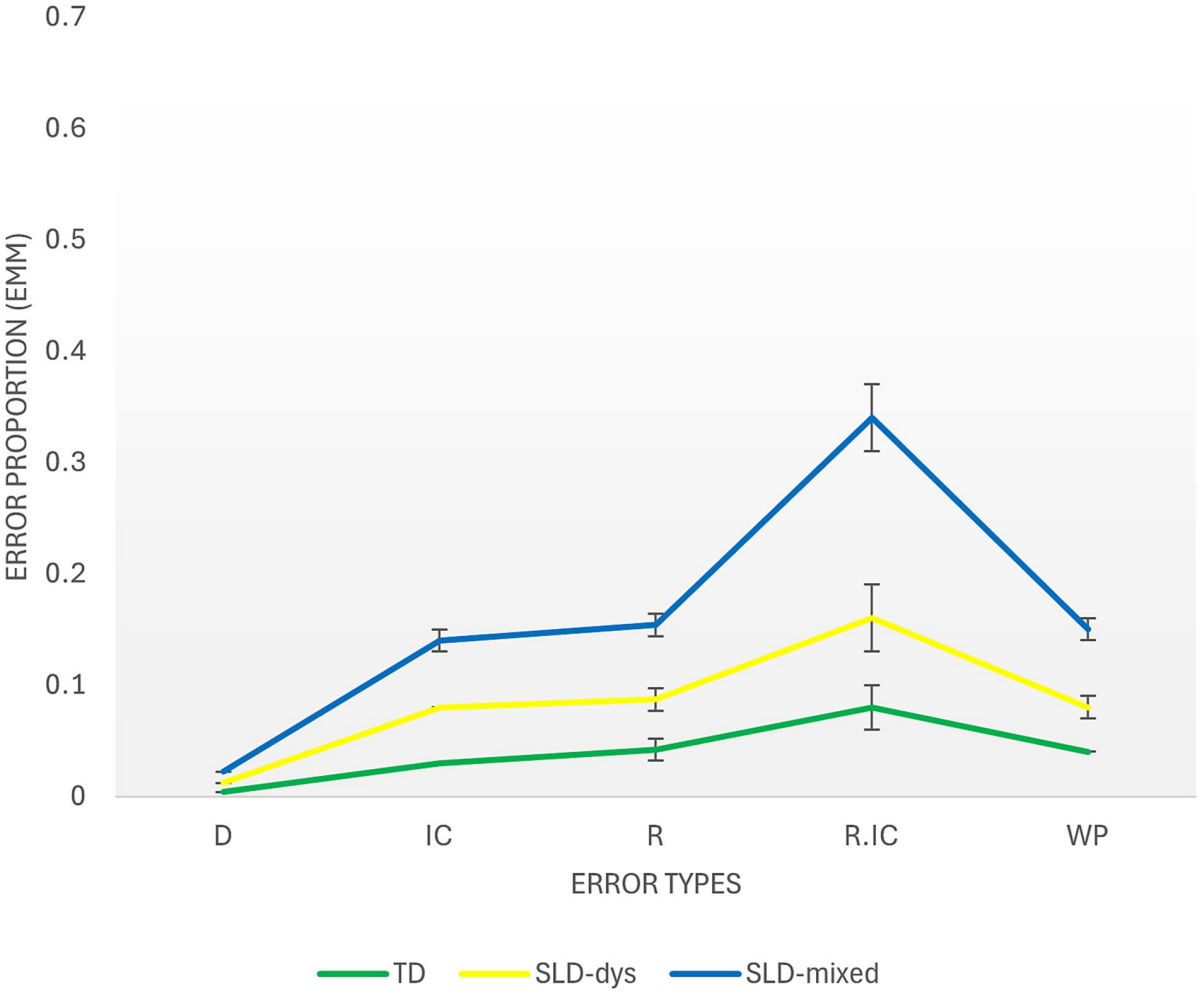
Analysis of error types in TD and SLD participants in the MatriKS 12+. The *x*-axis represents the different error types, and the *y*-axis shows the average percentage of error proportion committed by each group. TD, Typically Developing; SLD-Dys, Specific Learning Disorder-reading/writing subgroup; SLD-Mixed, Specific Learning Disorder-mathematics subgroup; M, Mean; SE, Standard Error.

The differences between groups in the proportion of error types in MatriKS 12 + is illustrated in Figure 4. At higher ages, the groups did not differ for the R (Wald χ^2^(2) = 4.74, *p* = 0.094). However, Age was a significant covariate (Wald χ^2^(1) = 5.78, *p* = 0.016), indicating that older adolescents made fewer repetition errors (b = −0.01; 95% CI [−0.01, −0.00]).

A significant main effect of Groups was found for WP errors (Wald χ^2^(2) = 12.77, p = 0.002). The SLD-Mixed group (M = 0.066; SE = 0.01) showed higher rate than both the TD group (M = 0.040; SE = 0.00, Wald χ^2^(1) = 11.76, *p* < 0.001) and the SLD-Dys group (M = 0.044; SE = -001, Wald χ^2^(1) = 7.16, *p* = 0.007). The TD and SLD-Dys groups did not differ significantly (*p* = 0.597). Age was not a significant covariate (*p* = 0.112).

Concerning D errors, a significant main effect of Group emerged (Wald χ^2^(2) = 6.73, *p* = 0.035), with the SLD-Mixed group (M = 0.014; SE = 0.00) showing more errors than the TD group (M = 0.004; SE = 0.00, Wald χ^2^(1) = 6.72, *p* = 0.010). While, the SLD-dys group (M = 0.009; SE = 0.00) did not differ significantly from either group. Age was not a significant covariate (*p* = 0.682).

A significant main effect of Group was also found for the IC errors (Wald χ^2^(2) = 16.15, p < 0.001). After adjusting for age, both the SLD-Dys group (M = 0.051; SE = 0.00, Wald χ^2^(1) = 4.88, *p* = 0.027) and the SLD-Mixed group (M = 0.065; SE = 0.01, Wald χ^2^(1) = 15.80, *p* < 0.001) produced significantly more incomplete correlate errors than the TD group (M = 0.034; SE = 0.00). The SLD-Dys and SLD-Mixed groups did not differ significantly (*p* = 0.107). Age was not a significant covariate (*p* = 0.283).

A significant main effect of Group was found for the R/IC error (Wald χ^2^(2) = 8.62, *p* = 0.013), evidencing that the SLD-Mixed group (M = 0.181; SE = 0.03) produced significantly more R/IC errors than both the TD group (M = 0.075; SE = 0.02, Wald χ^2^(1) = 7.58, *p* = 0.006) and the SLD-Dys group (M = 0.084; SE = 0.03, Wald χ^2^(1) = 5.43, *p* = 0.020). The TD and SLD-Dys groups did not differ (*p* = 0.819). Age was not a significant covariate (*p* = 0.229).

### 3.4 Diagnostic predictivity of error patterns (objective 4)

In order to assess whether error type proportions could predict group membership (TD vs. SLD-Dys vs. SLD-Mixed group), multinomial logistic regression was performed. For MatriKS 4–11 version, the overall model was statistically significant, χ^2^(10) = 25.43, *p* = 0.005, indicating that the error patterns reliably distinguished between the three groups. The model explained 29.1 to 33.2% of the variance in group membership (Cox and Snell *R*^2^ = 0.291; Nagelkerke *R*^2^ = 0.332) and correctly classified 62.2% of cases. Examination of the likelihood ratio tests revealed that WP errors significantly contributed to the model, (χ^2^(2) = 7.40, *p* = 0.025). The associated odds ratio was extremely small (Exp(b) = 1.329E-15; 95% CI = [1.339E-28, 0.013]). This reflects the scaling of the predictor, as error types were expressed as proportions bounded between 0 and 1, rather than indicating implausibly large or unstable effects. Accordingly, the magnitude of the odds ratio should be interpreted with caution, focusing on its direction and statistical significance. In this context, higher proportions of WP errors were associated with a lower likelihood of belonging to the reference group (TD), highlighting their diagnostic relevance. Othererror types did not reach statistical significance (R: χ^2^(2) = 4.60, *p* = 0.100; D: χ^2^(2) = 2.20, *p* = 0.333; IC: χ^2^(2) = 1.53, *p* = 0.466; R/IC: χ^2^(2) = 0.63, *p* = 0.730). Specifically, the model showed high classification accuracy for TD children (88.9%) and moderate accuracy for the reading/writing subgroup (SLD-Dys, 57.1%), but performed poorly in identifying the mathematics subgroup (SLD-Mixed, 11.8%).

Considering MatriKS 12 + version, the multinomial logistic regression model was also statistically significant (χ^2^(10) = 25.61, *p* = 0.004), with the error patterns distinguishing between groups. The model explained 25.8 to 29.1% of the variance (Cox and Snell *R*^2^ = 0.258; Nagelkerke *R*^2^ = 0.291) and correctly classified 60.5% of cases. In this model, likelihood ratio tests indicated that IC errors were the only significant predictor (χ^2^(2) = 10.53, *p* = 0.005). The corresponding odds ratio was small (Exp(b) = 2.229E-12;95% CI = [1.099E-22, 0.045]). This again reflects the scaling of the predictor, as error types were expressed as proportions bounded between 0 and 1, rather than indicating implausibly large or unstable effects. Accordingly, the magnitude of the odds ratio should be interpreted with caution, focusing on its direction and statistical significance. In this context, higher IC error proportions were associated with a lower likelihood of belonging to the reference group (TD). Other error types did not significantly contribute to the model (R: χ^2^(2) = 1.07, *p* = 0.585; D: χ^2^(2) = 2.12, *p* = 0.347; WP: χ^2^(2) = 1.63, *p* = 0.442; R/IC: χ^2^(2) = 2.95, *p* = 0.228). Classification accuracy was highest for TD children (83.3%), moderate for the SLD-Mixed subgroup (53.8%), and poor for the SLD-Dys subgroup (33.3%). Overall, these findings suggest that, when proportion-based predictors are used, odds ratios may assume extreme values due to scaling, and therefore should be interpreted primarily in terms of direction and statistical significance rather than magnitude.

## 4 Discussion

The aim of the present study was to investigate FI in children and adolescents with SLDs using MatriKS, a novel computerized adaptation of Raven’s Progressive Matrices (de Chiusole et al., 2024; de Chiusole et al., 2025). More specifically, we aimed at investigating (1) the convergent validity of MatriKS compared to Raven’s CPM and SPM, (2) possible differences in the overall accuracy on MatriKS between typically developing (TD) children and those with SLDs (distinguishing between SDL-Dys and SLD-Mixed subgroups), (3) whether distinct error patterns on MatriKS would emerge between healthy controls and specific learning disorders groups (i.e., SLD-Dys and SLD-Mixed), (4) the predictive value of error patterns on MatriKS in respect to the different groups (i.e., TD, SLD-Dys, and SLD-Mixed).

Regarding the first objective, the results of the correlation analyses demonstrated moderate convergent validity of MatriKS across developmental stages (Post, 2016), while also highlighting potential influences of administration format and sample characteristics on the strength of these associations. In particular, correlations were significant and positive both between MatriKS 4–11 and Raven’s CPM, and between MatriKS 12 + and Raven’s SPM. These results are in line with a previous study (de Chiusole et al., 2025), which investigated the validity of MatriKS, and support the utility of this tool for the assessment of fluid intelligence in children and adolescents. Moreover, the fact that it is a computerized instrument that automatically records and collects numerous information on the test-taker’s performance suggests that it may represent an efficient and information-rich alternative compared to the paper-and-pencil versions of Raven’s matrices (Mead and Drasgow, 1993; Witt et al., 2013).

Regarding the second aim, the results of the ANCOVAs showed differences in the overall accuracy on MatriKS between TD children and those with SLD, with the control group scoring generally higher than the SLD groups, but with a few differences depending on MatriKS version (i.e., MatriKS 4–11 and MatriKS 12+). In particular, when considering younger children who completed the MatriKS 4–11 version, the results showed that the control group performed significantly better in respect to both the SLD-Dys group and the SLD-Mixed group, which, on the contrary, did not differ between them. Given the scarcity of studies investigating the performance of children with SLDs on Raven’s Matrices, comparisons between the present results and previous findings are limited. On the one hand, the fact that typically developing children scored higher on MatriKS 4–11 compared to the SLD groups seems in contrast with the study by Bayliss et al. (2005), who found higher accuracy on CPM in children with learning difficulties compared to TD children; however, the trend they found was not significant. The present findings also contrast with previous research using the Wechsler scales that again found similar or even better performance of individuals with SLDs on visuo-perceptual tasks. For instance, Scorza et al. (2023) found a higher performance of participants with SLDs on the PRI index (assessing visuo-perceptual and non-verbal fluid reasoning) of the WAIS-IV compared to controls. However, the differences with the control group were significant only for participants with developmental dyslexia, and not for participants with mixed SLDs, suggesting that performance on visuo-perceptual tasks may vary depending on the area of academic impairment (Scorza et al., 2023), as will be discussed further below. Moreover, the age of the participants in both studies (Bayliss et al., 2005; Scorza et al., 2023) was different from the present study, as they included older children and university students respectively, and it could be hypothesized that differences in fluid abilities may narrow or change with age, thanks to the development of working memory and processing speed abilities, which are closely related to FI development (Fry and Hale, 1996, 2000). In line with that, in the present study, the analyses on older participants showed partially different results (see below).

On the other hand, our findings align with Poletti’s study (Poletti, 2016), which showed that children with SLD, compared to controls, performed worse, even if nearly on average, on the PRI subtests of the WISC-IV, including Matrix Reasoning subtest, although there were further differences when distinct subtypes of SLDs were considered. Overall, these contrasting findings on visuo-perceptual abilities in individuals with SLDs may be due to the great heterogeneity that characterizes their cognitive profiles (Cornoldi et al., 2019b; McGrath et al., 2019; Menghini et al., 2010; Pennington, 2006; Pennington et al., 2012; Poletti, 2016; Scorza et al., 2023; Toffalini et al., 2017; Willcutt et al., 2013), and to the methodological differences of the studies, which may result in different findings depending on the participants’ age or area of impairment, and on the task used.

As for the effect of age in the younger group (MatriKS 4–11), the ANCOVA showed that it was a significant covariate, revealing that greater age was associated with higher fluid abilities. These results are consistent both with models of FI development (Fry and Hale, 2000; McArdle et al., 2002) and with previous studies investigating age-related differences on Raven’s test (e.g., Gunn and Jarrold, 2004), which argue that performance tends to improve with age during childhood.

Regarding older participants who completed the MatriKS 12 + version, results showed that TD participants again obtained higher scores on the task compared to SLD groups, even if the differences were significant only in respect to the SLD-Mixed group. Moreover, a significant difference also emerged between SLD groups, with the SLD-Dys group showing a significantly better performance on MatriKS compared to the SLD-Mixed group. This is in line with theories and studies that highlight the cognitive heterogeneity of SLDs (Cornoldi et al., 2019b; McGrath et al., 2019; Menghini et al., 2010; Pennington, 2006; Pennington et al., 2012; Poletti, 2016; Scorza et al., 2023; Toffalini et al., 2017; Willcutt et al., 2013). In particular, the present findings are consistent with studies (e.g., Poletti, 2016; Toffalini et al., 2017) that investigated cognitive functioning in children and adolescents with different subtypes of SLDs using the WISC-IV and found a lower performance on the PRI in individuals with mathematical disorder, isolated or combined with other SLDs, compared to participants with other learning disorders, especially reading disorder.

Regarding age, contrary to the younger group, in MatriKS 12 + its effect was not significant. This may be linked to the fact that fluid abilities tend to increase rapidly particularly during childhood, then more gradually in adolescence, before stabilizing in early adulthood and starting to decline in older age (Fry and Hale, 2000; McArdle et al., 2002). Therefore, even if an increase in FI is still expected in adolescence, the effect of age may be less evident in older participants compared to younger ones, explaining the non-significant result found in the present study. This could be further amplified by the fact that the age range of this older group of participants completing MatriKS 12 + was larger (12 to 19.11 years) than the group of children completing MatriKS 4–11 (7.8 to 11 years).

Finally, it should be noted that, even if for the present study we considered the *raw scores* obtained by participants on Raven’s Matrices (i.e., CPM and SPM), the scoring based on Italian *normative data* of the CPM and SPM tasks, based on age, showed that the performance of all participants, including those with a diagnosis of SLD, fell within the normative range. This confirms that children and adolescents with SLD do not generally have deficient visuo-perceptual and fluid reasoning abilities, as also demonstrated by previous studies (e.g., Bayliss et al., 2005; Cornoldi et al., 2014, 2019a; Iaia et al., 2025; Poletti, 2016; Scorza et al., 2023; Toffalini et al., 2017) and in line with SLD diagnostic criteria (DSM-5 [American Psychiatric Association, 2013], ICD-10 [World Health Organization, 1992]) that exclude the presence of intellectual disability for the diagnosis. However, the results of the present study suggest that, upon closer investigation, in comparison to TD peers, children and adolescents with SLD, and especially those with mathematical disorder, may show lower levels of fluid abilities, as assessed by Raven-like tasks, even if they fall within the average range. Given that FI has been shown to support the development of crystallized intelligence and to predict achievements in several academic domains (Cattell, 1971, 1987; Droder et al., 2024; Green et al., 2017; Johann et al., 2020; Mano et al., 2018; Passolunghi et al., 2022; Peng et al., 2019), this suggests that investigating fluid abilities in this population should be considered important, as it may provide useful information to support their learning in prevention and treatment interventions.

Regarding the third objective, qualitative analysis of error patterns provided more in-depth information compared to overall accuracy scores alone. In the younger group (MatriKS 4–11), distinct error patterns emerged between diagnostic groups. Specifically, children in the SLD-Mixed group committed significantly more R errors compared to both TD children and those with SLD-Dys, and significantly more errors in the combined R/IC category compared to TD children. Regarding children with SLD-Dys, they showed a higher frequency of WP errors compared to TD controls. Moreover, a marginally significant effect emerged for IC errors, with the SLD-Dys group tending to commit more IC errors compared to the TD group. In the older group (MatriKS 12+), results showed partially different patterns. The SLD-Mixed group continued to show greater difficulties, committing significantly more WP, D, IC, and R/IC errors compared to TD controls. Furthermore, for IC and R/IC errors, the SLD-Mixed group showed significantly worse performance also compared to the SLD-Dys group. The latter committed more IC errors compared to TD, but did not show significant differences in other error types. These results confirm that qualitative analysis of performance, which extends beyond simple global accuracy scores, can reveal differences in underlying cognitive processes that are clinically significant yet remain hidden in traditional quantitative analysis, as emphasized by Kunda et al. (2016). In particular, IC and R error patterns seem to reveal specific vulnerabilities in children and adolescents with SLDs, and especially in those with mathematical impairments. The nature of these vulnerabilities may be linked to the specific cognitive processes that, when impaired, lead to these particular error patterns. Indeed, different error types differ in severity and in the cognitive processes they implicate.

For instance, IC errors, which occur when the selected distractor approximates the correct answer but differs in a perceptual feature (orientation, direction, number of elements; Raven et al., 2003) and are therefore considered the least severe, may reflect a failure in visuoperceptual skills related to the orientation and direction of elements (Kunda et al., 2016), but also difficulties in other high-order functions such as working memory and attentional control. Regarding working memory, Belacchi and Cornoldi (2009) documented that IC errors increase with age in the general population as children attempt more sophisticated reasoning strategies that place greater demands on working memory capacity. Similarly, Carpenter et al. (1990) showed that in the Advanced Progressive Matrices (Raven, 1965) good performance is associated with the ability to actively maintain task resolution rules in memory, and with effective control and monitoring in the use of rules and information present in the problem. This underscores the crucial importance of working memory for matrix resolution, in line with studies supporting the presence of a strong link between working memory, especially in its visuo-spatial components and FI (Fry and Hale, 1996, 2000; Rodriguez-Martínez et al., 2023). Moreover, it suggests that the presence of working memory deficits, which are indeed common in SLD population (Cornoldi et al., 2014, 2019a; Iaia et al., 2025; Poletti, 2016; Scorza et al., 2023; Toffalini et al., 2017) could prevent actively maintaining all necessary rules for the resolution of the matrix or lead to forgetting them at the crucial moment when distractor characteristics must be compared with matrix features to select the correct alternative, leading to the selection of a distractor which is similar, but not identical, to the correct one (IC error). In addition to that, IC errors could reflect deficits in attentional systems (Bolster et al., 1986; Crisci et al., 2021), specifically the Central Executive (Baddeley and Hitch, 2000) and the Supervisory Attentional System (Shallice, 1988), which could compromise strategy selection, maintenance of activation of relevant information, inhibition of irrelevant material, and the constant “updating” of working memory that must replace obsolete information with new and more pertinent material. Finally, another aspect that may underlie IC errors is related to impulsivity (Donfrancesco et al., 2005; Purvis and Tannock, 2000; Venkatesan and Lokesh, 2019), as higher levels of impulsivity could lead the individual to rapidly select among the first similar elements without systematically exploring all available options. Concerning R errors, it is known that they occur when the selected distractor replicates an element adjacent to the blank space, representing an inadequate intrusion response caused by dependence on the matrix space (Belacchi et al., 2012; Kunda et al., 2016). This pattern suggests a failure to grasp relationships between elements across rows and/or columns of the matrix: it is as if the matrix contained too much information, leading attention to focus on elements immediately surrounding the missing cell rather than on the overall relational structure (Carpenter et al., 1990). Belacchi and Cornoldi (2009) showed that in typical development, R errors increase until approximately age 7 and then decline progressively. This pattern might reflect the fact that in some matrices the choice of the alternative adjacent to the target is adequate, leading younger children to inappropriately generalize this strategy. With maturation and development of working memory capacity, children become progressively capable of maintaining a holistic view of the matrix structure. The persistence of elevated R errors in children with SLDs, even in the older age range, could suggest developmental delay or an atypical trajectory. This is consistent with evidence from clinical populations: Belacchi et al. (2012) found R errors more common in children with Down Syndrome, interpreting them as evidence of reduced processing capacity, similar to studies on neurological damage, psychiatric disorders, and intellectual disability (Goharpey et al., 2013). The most plausible explanation again concerns the crucial role of working memory, and especially of visuo-spatial working memory, which has been shown to be linked to matrix resolution tasks (Rodriguez-Martínez et al., 2023): weaker and deficient capacities may lead to poorer performance with a singular error pattern distant from that observed in typically developing peers of matched mental age (Carpenter et al., 1990).

The joint presence of IC and R errors in SLD suggests that difficulties encompass both the capacity to maintain and manipulate information in working memory with adequate executive control (IC) and the ability to maintain an integrated and holistic representation of complex visual information while avoiding cognitive overload (R). These vulnerabilities extend beyond domain-specific academic skills to encompass more general aspects of executive functioning and information processing.

Finally, it is particularly interesting to note how error patterns differ between SLD subgroups. The SLD-Mixed group, which includes children with isolated dyscalculia or in combination with reading/writing difficulties, showed more pervasive difficulties, with higher frequencies of R errors in the younger group and of WP, D, IC, and R/IC errors in the older group. In contrast, the SLD-Dys group showed a more circumscribed pattern of difficulties, with greater WP errors in the younger group and IC errors in the older group.

Regarding the fourth objective, multinomial logistic regression analyses examined whether error patterns could predict membership in the different groups (i.e., TD vs. SLD-Dys vs. SLD-Mixed group), in order to see if qualitative aspects of performance could help distinguish between diagnostic groups highlighting possible specific pattern responses by error type and age.

Overall, the models showed moderate classification accuracy in both age ranges and indicated that only certain types of errors meaningfully contributed to group differentiation. Moreover, the error types that emerged as significant predictors differed across developmental stages. In particular, WP errors resulted more discriminative in younger children, while IC errors were more discriminative in adolescents, possibly reflecting developmental changes in problem-solving strategies underlying matrix reasoning and distinct cognitive profiles associated with different SLD subtypes across age ranges. The discriminative capacity of WP errors in the younger group might reflect fundamental misunderstanding of the logical relationships in the matrix, which are more evident when children are still developing their fluid reasoning abilities and systematic problem-solving strategies. With development, most children, both TD and with SLD, improve in understanding the basic principles of matrices, making WP errors less discriminative. In contrast, IC errors become more discriminative in adolescents, likely because they reflect more subtle difficulties in working memory and executive control that persist or become more evident when tasks increase in complexity. In adolescents, where greater maturation of basic cognitive abilities is expected, the persistence of elevated IC errors might signal more persistent vulnerabilities in visuospatial working memory that continue to impact complex reasoning despite the development of compensatory strategies in other areas. The extremely small odds ratios observed in the multinomial logistic regressions reflect the proportional method used to calculate error types. While the magnitude of these estimates should be interpreted cautiously, they highlight the strong diagnostic relevance of specific error patterns: higher proportions of WP errors in younger children and IC errors in adolescents were strongly associated with a lower likelihood of belonging to the TD group, supporting their role as sensitive indicators of SLD subtypes across developmental stages. Whereas younger children’s performance is better characterized by errors stemming from incomplete mastery of logical principles, adolescents’ performance appears more shaped by the efficiency of executive and working memory processes. This distinction supports the broader interpretation that SLD subtypes may be associated with age-specific cognitive signatures, and that fine-grained performance metrics, such as error patterns, can provide valuable complementary information beyond overall accuracy.

## 5 Clinical implications

The current findings have important clinical implications, highlighting the importance of integrating qualitative analysis into the assessment of children with suspected or diagnosed SLD. First, examining error patterns in FI tasks such as MatriKS or Raven’s Matrices can help identify specific cognitive vulnerabilities, particularly in working memory and visuospatial processing, that are highly relevant for individualized intervention planning. For example, children who frequently commit Incomplete Correlate (IC) errors may benefit from interventions aimed at strengthening visuospatial working memory and enhancing their ability to hold and integrate multiple elements of a reasoning problem. Conversely, high rates of Repetition (R) errors may indicate a tendency to rely on locally salient features rather than on the underlying matrix structure, suggesting the need for interventions that foster systematic, global analysis and metacognitive strategy use.

Second, the differentiation of SLD-Dys and SLD-Mixed subgroups observed in this study suggests that intervention should be tailored to cognitive subtypes. Children in the SLD-Mixed group showed the most pervasive inefficiencies, characterized by elevated R errors in younger children and multiple error types (WP, D, IC, R/IC) in adolescents, consistent with previous research showing more widespread deficits in working memory, processing speed, and perceptual reasoning in mathematical disorders (Poletti, 2016; Toffalini et al., 2017). Children with SLD-Dys, in contrast, exhibited a more circumscribed pattern (higher WP errors in younger children; increased IC errors in adolescents), indicating more selective weaknesses in logical reasoning and visuospatial working memory. These differences underscore the need for subtype-specific intervention approaches: targeted reasoning and visuospatial WM support for SLD-Dys, and broader cognitive remediation and strategy training for SLD-Mixed.

Finally, the computerized nature of MatriKS, which enables the automated capture of accuracy, response latencies, and error types, can reduce examiner bias, enhance scoring precision, and provide an information-rich performance profile, useful to improve the accuracy and ecological validity of the assessment and the personalization of interventions.

## 6 Limitations and future directions

Several limitations should be acknowledged when interpreting these findings. First, although adequate for exploratory analyses, the sample size was modest once stratified by age group and diagnostic subtypes, which likely reduced statistical power. Replication with larger samples is essential, particularly to validate subgroup differences and predictive models of diagnostic classification.

Second, although the SLD subgroups were clinically meaningful, they remained heterogeneous. The subdivision into SLD-Dys and SLD-Mixed provided a more refined perspective; however, further differentiation, in accordance with diagnostic criteria from the ICD-10 and DSM-5, among isolated dyslexia, dysorthography, dyscalculia, and mixed profiles, would allow for a clearer delineation of their characteristic features and functioning profiles (American Psychiatric Association, 2013; World Health Organization, 1992). Previous evidence demonstrating heterogeneous cognitive profiles within these subtypes suggests that even more granular subgrouping could reveal more specific and informative error patterns (Cornoldi et al., 2019b; McGrath et al., 2019; Menghini et al., 2010; Pennington, 2006; Pennington et al., 2012; Poletti, 2016; Scorza et al., 2023; Toffalini et al., 2017; Willcutt et al., 2013).

Third, the cross-sectional design limits conclusions about developmental trajectories. Longitudinal research is needed to determine whether error profiles change with age, whether the reduced differences observed in adolescence reflect true convergence or compensatory strategies, and whether early error patterns predict later academic outcomes. Such studies may help identify early cognitive markers of persistent versus remitting learning difficulties.

Finally, the study focused exclusively on TD and SLD groups. Comparing error profiles across broader neurodevelopmental conditions (e.g., ADHD, ASD, Intellectual Disability) would help establish whether certain error types (e.g., R errors reflecting attentional dysregulation; WP errors reflecting difficulties in abstraction and rule extraction) are specific to SLD or represent transdiagnostic cognitive markers. Moreover, potential moderating variables such as socioeconomic background, linguistic experience, comorbidities, and prior intervention history should be examined to better understand individual variability in fluid reasoning performance. Furthermore, another aspect to consider is the possibility of extending the analysis of errors to include skipped responses, which may provide additional information on participants’ engagement, perceived task difficulty, or fatigue. Future studies could integrate these data to enhance the sensitivity of MatriKS in detecting individual differences in performance.

In addition to these future directions, the current study opens up further lines of research. First, given that response times constitute a valuable source of information on cognitive performance, a systematic examination of the relationship between response times and error patterns would offer further insights into the underlying cognitive processes or individual characteristics (e.g., tendencies to impulsivity). Second, given that the matrices in MatriKS are generated according to different rules (e.g., logical and visuo-spatial rules; Brancaccio et al., 2025), it would be important to investigate potential interactions between error type and matrix characteristics, such as the type of rule required to answer an item, the number of rules involved, and the perceptual or logical complexity of the elements. For instance, some items require a single rule, such as a color change across cells, while others involve multiple rules, for example, a combination of color, shape, and orientation changes simultaneously. Items may also differ in perceptual complexity, such as figures with multiple visual features (color, shape, size), or in logical complexity, requiring deduction or integration of multiple conditional rules. Previous literature suggests that the number of matrix elements, the number and complexity of rules, and the perceptual characteristics of the elements all contribute to item difficulty and cognitive load (Primi, 2001). Therefore, examining how specific error types relate to these matrix features could provide insight into the underlying cognitive processes involved in problem-solving and fluid intelligence abilities. Moreover, evidence indicates that certain errors are more frequent in individuals with lower abilities, while other errors tend to occur more frequently in those with higher abilities (Babcock, 2002; Bromley, 1953; Goodman, 1977). This suggests that it would also be interesting to analyze possible interactions between error type and the participant’s ability level. Third, future studies should investigate the association between fluid intelligence performance on the MatriKS task, considering both total accuracy and qualitative error types, and performance on measures of cognitive abilities, academic skills (reading, writing, and arithmetic), and cross-domain learning skills such as attention and memory. This would help determine whether specific error profiles contribute to academic and general cognitive functioning, strengthening the predictive validity of qualitative error analysis. Fourth, performing a cluster analysis on error types to determine whether MatriKS error patterns align with diagnostic classifications constitutes a valuable contribution for future research.

Finally, one promising extension of the current framework involves the use of polytomous Knowledge Space Theory (KST; Stefanutti et al., 2020a; Stefanutti et al., 2020b) models or, more generally, probabilistic latent class models, for the analysis of errors. Although grounded in rigorous mathematical foundations, these models provide qualitative feedback that can be highly informative in clinical contexts. In particular, they are not limited to identifying which tasks or items an individual can successfully complete, but can also characterize the types of errors made. Such detailed information can be leveraged to enrich individualized training or intervention plans, offering a more nuanced understanding of an individual’s knowledge state and learning needs. By capturing both successful responses and systematic error patterns, probabilistic KST models provide a bridge between quantitative assessment and qualitative insight, enabling practitioners to design tailored educational or therapeutic strategies based on the specific cognitive profile of each individual.

## 7 Conclusion

This study shows that incorporating qualitative error analysis into FI assessment provides critical insights into the cognitive processes and difficulties (e.g., in working memory, executive control, and visuospatial processing) underlying learning difficulties, which would otherwise remain hidden through traditional scoring focusing solely on overall accuracy. These cognitive vulnerabilities in SLDs appear to change across development, with different types of errors becoming diagnostically informative at different ages, highlighting the importance of age-appropriate assessment approaches. Moreover, this qualitative evaluation could have important implications also for treatment interventions, supporting more targeted and individualized remediation strategies that focus not only on academic skills but also on the cognitive abilities that sustain learning. In this context, the computerized format of MatriKS facilitates this qualitative evaluation by automatically capturing detailed performance data with enhanced precision and efficiency.

## Data Availability

The raw data supporting the conclusions of this article will be made available by the authors, without undue reservation.
